# Potential of Graphene Doping Towards Superlubric Performance of Additively Manufactured Structures

**DOI:** 10.3390/ma18122730

**Published:** 2025-06-10

**Authors:** Pinelopi Katsivardi, Nikolaos Koutroumanis, Alexandros E. Karantzalis, Pantelis G. Nikolakopoulos, Konstantinos G. Dassios

**Affiliations:** 1 Department of Chemical Engineering, University of Patras, Caratheodory 1, 26504 Patras, Greece; up1073054@ac.upatras.gr; 2 Adrine, Patras Science Park, Stadiou Str., 26504 Patras, Greece; nkoutrou@adrine.gr; 3 Department of Materials Science and Engineering, University of Ioannina, 45110 Ioannina, Greece; akarantz@uoi.gr; 4 Department of Mechanical Engineering and Aeronautics, University of Patras, 26504 Patras, Greece; pnikolakop@upatras.gr

**Keywords:** nanocomposites, graphene, superlubricity, friction

## Abstract

Significant improvements in the tribological performance of graphene-doped additively manufactured structures are reported, with absolute values of friction coefficients reaching 0.09 corresponding to ca. 70% decreases from plain/un-doped samples. The findings highlight an impressive potential of the nanocarbon variant, to endow superior tribological performance to polymers, bringing them a step closer to the ideal superlubric regime. Such structures of intrinsic superlubric performance are envisioned as viable candidates for the containment of great amounts of energy, currently wasted as friction in a plethora of applications, hence also promoting an ecologically sustainable development. Indications that superlubricity is greatly promoted by nanocarbons, especially by the two-dimensional variant of graphene with excellent response in shear action, are investigated in combination with the effect of surface topography, for the investigation of the tribological performance of three-dimensional structures with geometric surface patterning, additively manufactured from graphene-doped polymers. Spectroscopic, mechanical, and microstructural characterization of plain polymer-based samples and their graphene-enhanced nanocomposite counterparts was followed by tribometric measurements for the establishment of the evolution of the friction coefficient on a certified commercial tribometer operating under the ball-on-disk configuration as well as on a conceptual purpose-built setup. The individual and combined effects of nanomaterial presence and patterning are reported, and the influence of manufacturing-prone micropatterning is examined.

## 1. Introduction

Around one-third of the world’s primary energy production is lost due to friction in applications involving moving parts [1]. In all its forms, energy is intrinsically linked to industrial production, the sustainable development of economy, and everyday life. Our ever-increasing dependence on energy leads not only to source exhaustion, but also to environmental concerns, most critical of which is global warming. Hence, from an ecological and societal standpoint, the need to reduce energy consumption, and especially loss, is increasingly imminent. One of the biggest energy sink phenomena is friction, a natural occurrence that depletes large amounts of precious energetical input as futile heat while promoting material wear, hence also mechanical damage and component failure in industrial equipment; conditions which, in turn, can put human life, the environment, and equipment at great risk. Reduction of friction by small percentages can bring significant benefits in energy conservation, prolonged machine life, human safety, and environmental protection. Conventional methods of friction reduction, such as liquid lubrication, present limitations, particularly under demanding service conditions of high mechanical and thermal loads. Moreover, the disposal of such lubricants after the end of their lifespan raises toxicity and environmental concerns [2]. On such grounds, tribological research is currently exploring novel ways for minimizing friction and its effects.

One of the most prominent contemporary paths for friction minimization is superlubricity, the state of motion where friction between two surfaces nearly vanishes. It is practically achieved when the coefficient of friction, i.e., the parameter that quantifies the ease with which two surfaces slide against each other, equal to the ratio of the frictional force to the normal force, approaches values near 0.01 [3], while the coefficient normally varies between 0.3 and 0.6 for most materials. The concept of superlubricity was first confirmed experimentally in 1990, through tribometric measurements on mica surfaces [4]. At the nanoscale, superlubricity can be observed during the sliding of two atomically smooth crystal surfaces in incommensurate contact [5]. Various systems can exhibit superlubric behavior, from well-known multilayered materials such as graphite, to advanced composites and diamond-like carbons [6]. In the case of graphite, which constitutes one of the first materials to demonstrate superlubric features, friction between neighboring layers of carbon atoms arranged in a hexagonal lattice (like in graphene) can be almost eliminated when the relative orientation of the lattices, during sliding, is random. The requirement of incommensurate interaction for the manifestation of superlubricity highlights the importance of atomic structure and its orientation to the phenomenon [7]. Despite its promising prospects, the field of superlubricity still presents significant challenges, especially in the manifestation and maintenance of superlubric states at the macroscale, for the so-called structural superlubricity.

Two-dimensional (2D) nanomaterials, such as graphene, hexagonal boron nitride (h-BN), and transition metal carbides and nitrides (MXenes), recently gained increasing interest in tribology and have been explored as performance boosters for solid and liquid lubricants, as well as superlubric materials [8,9,10,11,12]. Specifically for graphene, Bustillos et al. reported that embedment of nanoplatelets (GNPs) can decrease a polymer’s coefficient of friction by 65%, pointing towards superlubric behavior [13]. Graphene nanofillers were also reported to be responsible for the reduction in the coefficient of friction in patterned PLA surfaces [14].

Surface patterning is, in fact, an independent lubrication method that has gained a lot of scientific interest in the last years. In essence, it involves the modification of the topography of a surface, to endow better tribological properties. These attribute both to the reduction of the contact area and to the trapping of wear debris within the pattern cavities, which can act as solid lubricants under subsequent dry sliding conditions [15]. Recent studies have also shown that when surface patterning is used to create geometric or biomimetic patterns, very low coefficients of friction can be achieved [14,16]. Surface patterning can conventionally be performed by techniques such as laser texturing, electrical discharge texturing, etching, and oxidation [17]. The recent vast developments in additive manufacturing technologies have also enabled the rapid and efficient manufacturing of patterns that exhibit very promising tribological behaviors [16].

As a fully customizable and diverse rapid prototyping technique that enables the creation of highly complex structures with good spatial resolution, three-dimensional (3D) printing opens new paths in the study of superlubricity, by adjustment of the shapes and patterns of 3D surfaces. Three-dimensional printing technologies can be categorized into seven main categories, namely, (i) vat photopolymerization, which uses an energy source like a laser beam to selectively cure a layer of photosensitive materials (e.g., stereolithography, digital light processing (DLP)); (ii) material extrusion, where materials are dispensed through an extrusion nozzle (e.g., fused deposition modelling (FDM)); (iii) powder bed fusion, where a powder bed is fused and sintered via a laser or an electron beam (e.g., selective laser sintering, selective laser melting (SLM)); (iv) binder jetting, which uses inkjet technology to selectively deposit a binder onto the powder bed; (v) material jetting, where liquid droplets are deposited on a substrate (e.g., inkjet printing (IJP)); (vi) sheet lamination, where the part is built by the lamination of sheet feedstock (e.g., laminated object manufacturing), and (vii) directed energy deposition, where powder or wire is combined with a laser to deposit materials onto a build plate [16]. Among this vast variety, SLM, DLP, IJP, and FDM are the most cited techniques, with the latter being probably the most widely applied 3D printing technology. Therein, polylactic acid (PLA) constitutes one of the most popular choices as filament material for FDM and other techniques, owing mainly to its low cost, relatively low glass transition (*T_g_*) and melting (*T_m_*) temperatures, high adaptability, and recyclability.

This study aims to investigate the combined effect of surface patterning and the presence of graphene nanoplatelets on the tribological behavior of 3D structures with geometrical patterns, fabricated by FDM from graphene-infused PLA filaments, with aspiration of achievement of superlubric conditions. The friction coefficients of both pure PLA and its graphene-enhanced counterpart were assessed using both a commercial tribometer and a conceptual custom purpose-built setup relying on ASTM D1894 specifications. The individual and combined effects of nanomaterial presence and patterning on the friction coefficients were investigated and reported, while the influence of manufacturing-prone micropatterning on the property was examined by considering different material deposition orientations on geometrically unpatterned structures. The results presented can assist tribologists and biotribologists in comprehending potential applications and optimization methods for PLA or other polymer-based materials, reinforced with treated and untreated graphene and its derivatives such as graphene oxide (GO), for example, on piston ring, or tribology gears as well as in load-bearing bone implants, application in which tribological aspects are vital.

## 2. Materials and Methods

### 2.1. Materials

In the current study, pure-PLA printing filaments and their GNP-enriched counterparts (“composite” filaments, commercial name GRAPHYLON 3D) were used for additively manufacturing structures that underwent tribological testing. Both filaments were acquired from FILOALFA Maip Compounding S.r.l. (Torino, Italy) and were 1.75 mm in diameter.

### 2.2. Fused Deposition Modeling (FDM)

**Figure 1:**
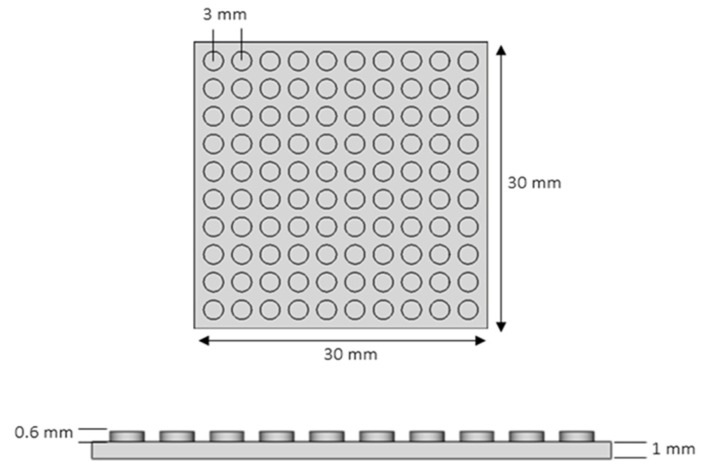
Dimensional characteristics of the samples with circular geometric surface patterning.

Based on previous tribological findings and knowledge [14,16], square samples of external dimensions of 30 × 30 mm^2^, with geometric surface patterns in the form of periodically-spaced short pillars of circular cross section, were designed and deposited. Structure design was performed on FreeCAD v. 0.21 software. Schematics of the geometrical pattern are depicted in Figure 1 along with dimensional characteristics.

**Figure 2:**
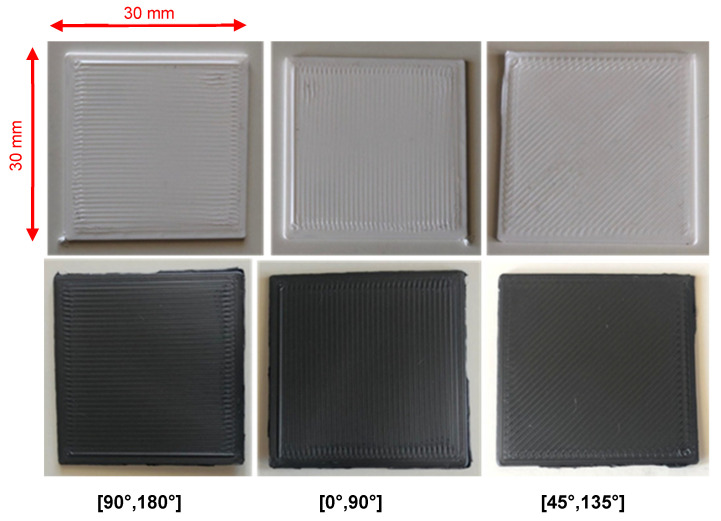
Plain structures with printer-induced surface micropatterning of orientations, from **left** to **right**, [90°, 180°], [0°, 90°], and [45°, 135°]. Top row samples were constructed from plain PLA, while bottom row ones, from graphene-doped PLA filaments, hence their dark color.

Plain, geometrically unpatterned samples of the same square section were also developed for tribological assessment of the printer-induced micropattern on the outer surface. The stereolithographic design files were imported into Ultimaker Cura 3D printing software (version 4.0, Ultimaker BV, Utrecht, The Netherlands) and converted to g-code to be further fed to the 3D printer. The software allowed the selection of the mode and the orientation of material deposition on the surface during printing (top surface skin pattern). In the current study, the native Zig Zag top surface mode was chosen, with varying orientations of [90°, 180°], [0°, 90°], and [45°, 135°], as demonstrated in Figure 2. The printing micropattern was not applied to 3D circular geometric structures and therefore did not affect the tribological behavior of those samples.

Sample deposition by FDM was performed on a Creality3D CR10-V2 printer (Shenzhen Creality 3D Technology Co., Ltd., Shenzhen, China) featuring a heated carborundum glass build-plate offering minimization of warping and ease of detachment of prints. Nozzle and plate temperatures were adjusted according to filament type. For PLA, nozzle temperature was set to 200 °C and bed temperature to 50 °C; respective values of 210 °C and 60 °C were used for PLA–graphene.

### 2.3. Spectroscopic and Morphological Characterization

The quality of the nanocomposite PLA-based starting materials/printing filaments was assessed through Raman spectroscopic testing, in an inVia™ confocal Raman microscope (Renishaw PLC, Gloucestershire, UK) using a solid-state laser excitation source, emitting at a wavelength of 532 nm. The most important spectroscopic parameter in the Raman spectra of graphene, is the intensity ratio of the D and G peaks, which is representative of the material’s degree of crystallinity and level of structural deficiency, while it also correlates to the number of graphenic layers in the corresponding nanoplatelets.

For the optical observation of the microstructure of the composite filament, small cross sections and longitudinal sections were encapsulated in typical mounting resin at a resin/hardener mixture of weight ratio of 35/100. After a hardening cycle of 48 h, the capsule surface was polished under rotational grinding, initially with 500# and 1000# grade grit paper to bring the graphene reinforcements in the filament to the sample surface, and ultimately with 2000# and 4000# grades for polishing elimination of surface roughness and optimization of the optical characterization aspects.

Microstructural analysis of as-printed structures was performed on a JSM 6300 scanning electron microscope (SEM, JEOL Ltd., Tokyo, Japan), equipped with a Link ISIS 300 X-ray Energy Dispersive Spectrometer (Oxford Instruments Plc, Abingdon, UK).

### 2.4. Mechanical Testing

The mechanical behavior of as-printed materials was studied under monoaxial tension in a hydraulic R858 Mini Bionix^®^ mechanical testing frame (MTS Systems, Eden Prairie, MN, USA). These experiments established the yield strength of the printed materials, which served as vital input for the determination of the required normal force range of the tribometric experiments conducted for the quantification of the surfaces’ friction coefficient. Young’s modulus of the materials was also calculated, which aided the quality assessment of the commercially available filaments.

Five rectangular tensile test specimens were 3D-printed for each of the two materials (PLA and composite filament) following the same cycle as the tribological structures. For comparison, additional sets of five similar specimens were fabricated by hot-pressing small pieces of the bulk materials, under 200 °C and 30 bar, for a duration of 90 s. All specimens were tested in uniaxial displacement-controlled tension, with a crosshead speed of 5 mm/s, according to the specifications of standard test method ASTM D-638-02 [18].

### 2.5. Tribometry

#### 2.5.1. Purpose-Built Conceptual Tribometer

**Figure 3:**
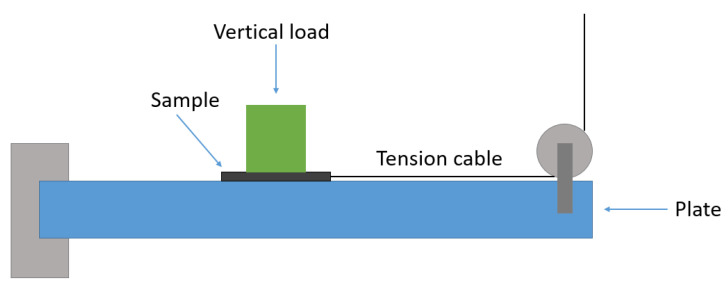
Outline of the conceptual purpose-built tribometric tester.

**Figure 4:**
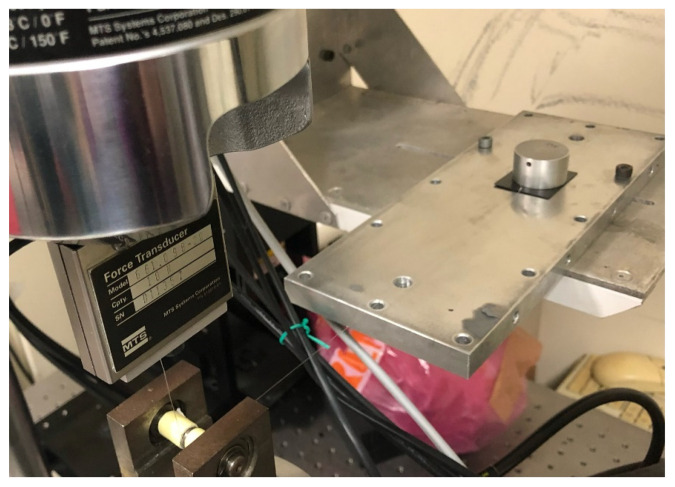
Tribometric test on the purpose-built jig. The cylindrical metallic weight is observable on the 3D-printed sample, while the latter is pulled across the metallic substrate.

Within the scope of the study, a custom tribometer was constructed in accordance with ASTM D1894 standard test method specifications, which refers to the determination of static and kinetic friction coefficients in sliding plastic films and sheeting [19]. The purpose-built instrument was driven by the Instron R858 Mini Bionix^®^ mechanical testing frame (Norwood, MA, USA), which was custom-equipped with a 10 N loadcell. The samples were attached to the loadcell with a metallic tension cable and slid under a vertical load of 5 N (by means of placement of a 500 g weight on top) across a metallic plate surface, as the moving (upper) crosshead of the tester was displacing, as shown on Figure 3. The crosshead displacement speed was set at 150 mm/min. In order to ensure a uniform stress distribution on the sample, the weight surface covered a large part of the sample surface, as shown in Figure 4. Measurements of frictional force versus displacement of each sample on the plate were obtained. Both plain samples with printer-induced surface micropatterns of different orientations and samples bearing the circular geometric pattern were tested in the conceptual jig. For repeatability, three tests were conducted for each material type.

#### 2.5.2. Ball-on-Disk Tribometer

Tribometric testing was also conducted for all samples (both those carrying the printer-induced surface micropatterns and those bearing the circular geometric patterns) on a commercial certified CSM ball-on-disk tribometer (CSM Instruments SA, Peuseux, Switzerland). Samples under investigation were placed on a round base inside the instrument’s main chamber, where a lever with a 6 mm diameter steel ball tip was put in contact with the specimens’ surface. Two linear variable differential transformer (LVDT) sensors recorded the minute variations of the lever during rotating and sliding of the tip on the surface under investigation and converted them to frictional forces. The ratio of the sensor signal (friction force) to the applied vertical load was then used for the calculation of the friction coefficient. Based on the mechanical testing findings (Section 2.4) and the condition that local stress levels should reach values marginally greater than the yield stress of the materials (55 MPa), a vertical load of 5 N was employed. The ball translated rotationally on the sample surface at a standard speed of 16 mm/s. Every test was performed at room temperature conditions (20 °C, 70% humidity) for a duration of 3600 s (60 min).

The tribological performance and experimental characterization of PLA–graphene does not lack scientific attention in the last years [20,21]. However, despite the promising tribological applications of PLA in specific applications, such as biocomposites, research on their wear behavior is still lacking [22]. 

## 3. Results and Discussion

### 3.1. Spectroscopic Characterization

**Figure 5:**
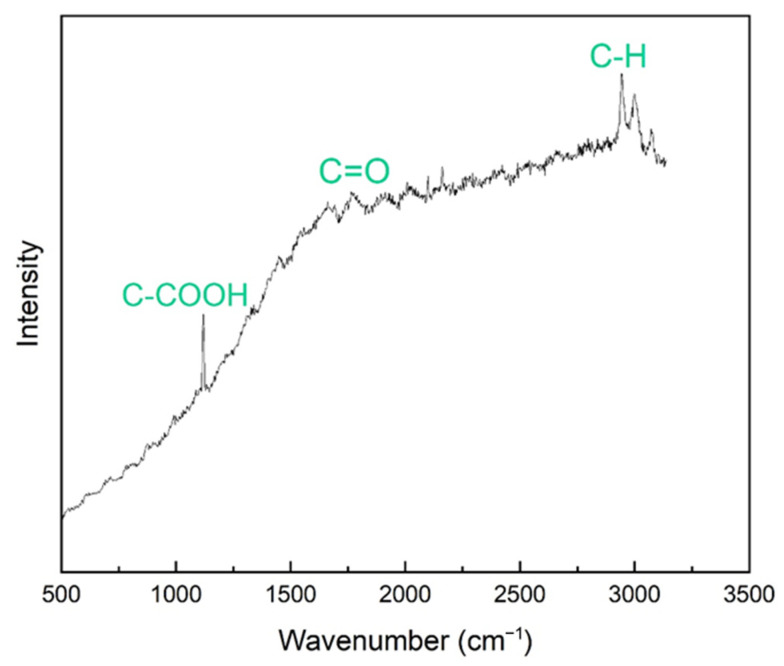
Typical Raman spectrum of PLA filaments.

**Figure 6:**
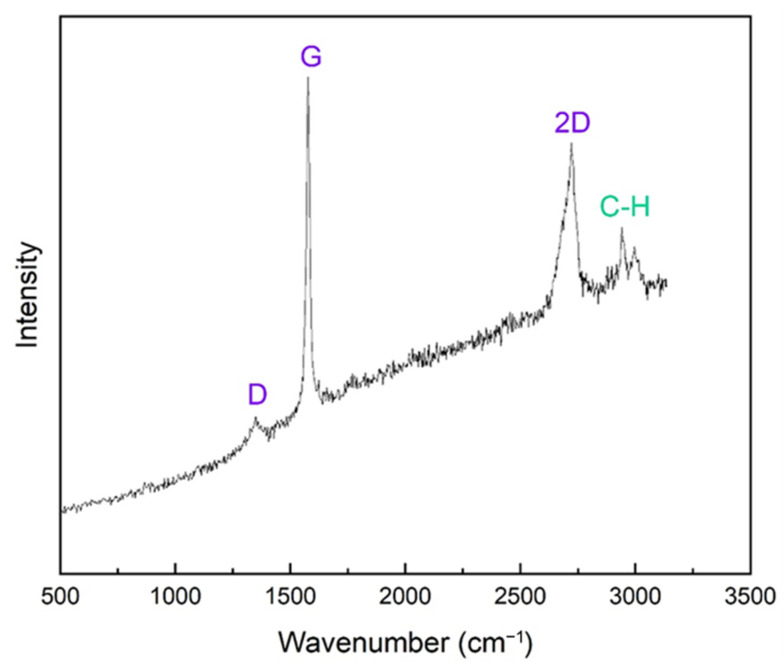
Typical Raman spectrum of composite PLA filaments with embedded graphene nanoplatelets.

The Raman spectroscopic response of the starting PLA filament material is presented in Figure 5, wherein characteristic peaks are observed at 2943 cm^−1^, 1767 cm^−1^, and 1119 cm^−1^ wavenumbers, attributable to the respective vibrations of carbon–hydrogen (C–H), carbonyl (C=O), and carboxyl (C–COOH) bonds. The corresponding typical Raman spectrum of composite PLA–graphene filaments is shown in Figure 6. The characteristic D, G, and 2D peaks are observed at wavelengths of 1350 cm^−1^, 1578 cm^−1^, and 2722 cm^−1^, respectively, in accordance with expectations for graphene presence. In addition, the peak corresponding to the C–H bond of PLA is observed at 2942 cm^−1^. The intensity ratio of the D and G peaks, I_D_/I_G_, is equal to 0.265, while that of the 2D and G peaks, I_2D_/I_G_, is equal to 0.856. These values indicate the multilayered nature of graphene nanoplatelets in the composite filaments. Moreover, the intensity ratios of the D and G peaks indicate high crystallinity and absence of significant lattice defects of the embedded platelets.

### 3.2. Microstructure

**Figure 7:**
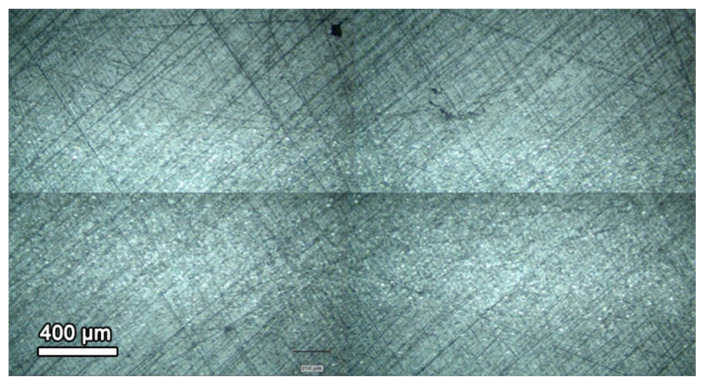
Internal structure of the composite PLA filament embedded with graphene nanoplatelets.

The typical internal microstructure of the composite PLA filament with embedded graphene nanoplatelets, as assessed by optical microscopy imaging, is shown in Figure 7. It can be observed that the distribution of the nanoplatelets is not uniform across the filament radius, and two GNP-rich zones are evident close to the central axis of the filament, which probably attribute to specifics of the material extrusion process in the final stage of filament production.

**Figure 8:**
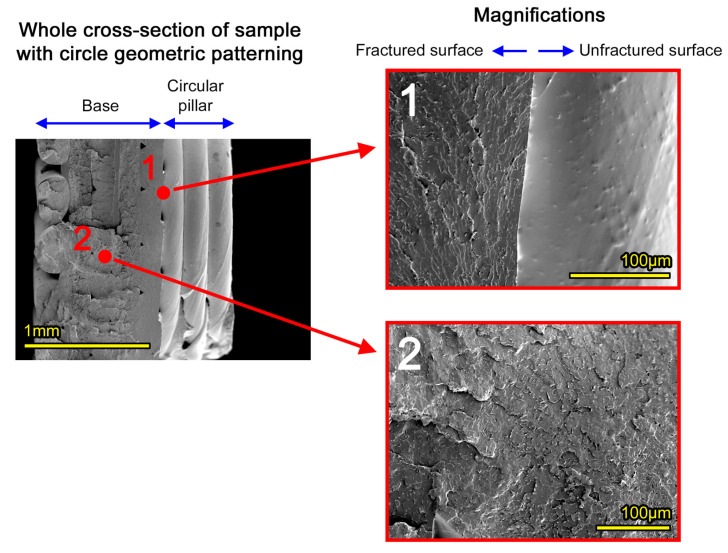
Internal microstructure of additively manufactured structures from PLA-GNP filaments, demonstrating homogeneous dispersion of the flakes in the polymer.

The internal morphology of as-printed graphene-doped structures was analyzed under SEM examination, and two typical micro-morphologies, collected at critical locations on the cross section, are presented in Figure 8. The first location (denoted by index 1 in the figure) corresponds to the interface between the fractured and unfractured part of the cross section. In the magnified micrograph on the right, the fractured region reveals the tips and sides of fractured graphene flakes as brighter points. The feature is absent on unfractured surfaces of the same image. The second micrograph shows a random location in the cross section, carrying the same speckle pattern with bright ends and sides of GNPs. Overall, the microstructure observed by the morphological analysis signifies a uniform dispersion and random orientation of flakes within the polymer, after its melting for deposition, and the absence of agglomerates.

### 3.3. Mechanical Testing

**Figure 9:**
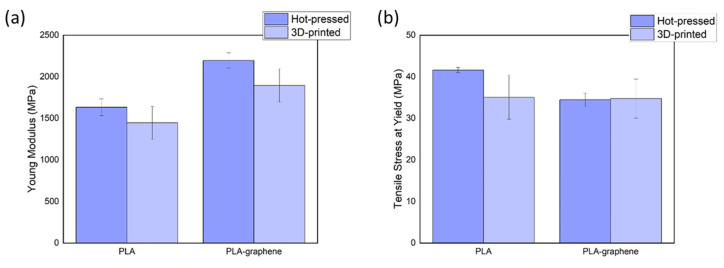
Comparisons of the materials’ main mechanical properties: (**a**) Young’s modulus and (**b**) yield strength.

The stress–strain responses of as-printed and hot-pressed specimens provided their primary mechanical properties, namely, Young’s modulus and tensile and yield strengths, as summarized in Table 1. It is observed that the addition of graphene brings a 31.1% increase in the elastic modulus of PLA for the 3D-printed samples and a 34.5% increase for their hot-pressed counterparts, as also plotted in Figure 9. It is worth mentioning that the slight divergence in mechanical properties’ values calculated from the two production methods is not surprising; since 3D-printed samples usually contain voids and defects, they are less compacted, and therefore, their mechanical response is not always directly comparable to those of the bulk fully dense material.

As previously mentioned, the establishment of the yield strength of materials was indispensable information for the tribometric tests, since the tensile stress at yield serves as the minimum contact pressure limit of the tribometer tip, as carried out within the framework of the current study. This is based on the fundamental requirement that the samples are required to marginally yield during testing to allow the achievement of the ultra-low frictional state [11,23]. Based on the results presented above, a contact pressure of 55 MPa was selected for the tribological measurements, common to both materials.

### 3.4. Tribometry

#### 3.4.1. Purpose-Built Conceptual Tribometer

**Figure 10:**
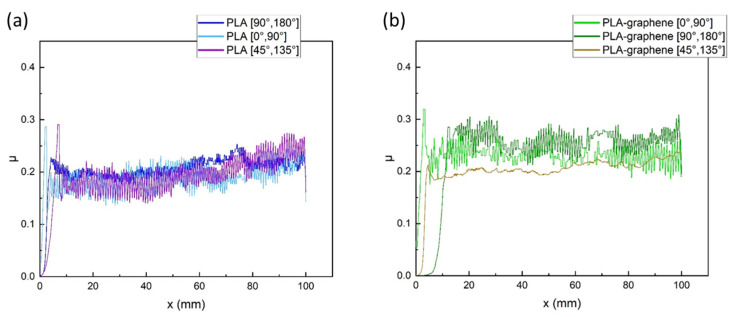
Coefficient of friction versus displacement for plain structures bearing only printer-induced surface micropatterning of different orientations: (**a**) pure PLA and (**b**) PLA–graphene composites.

**Figure 11:**
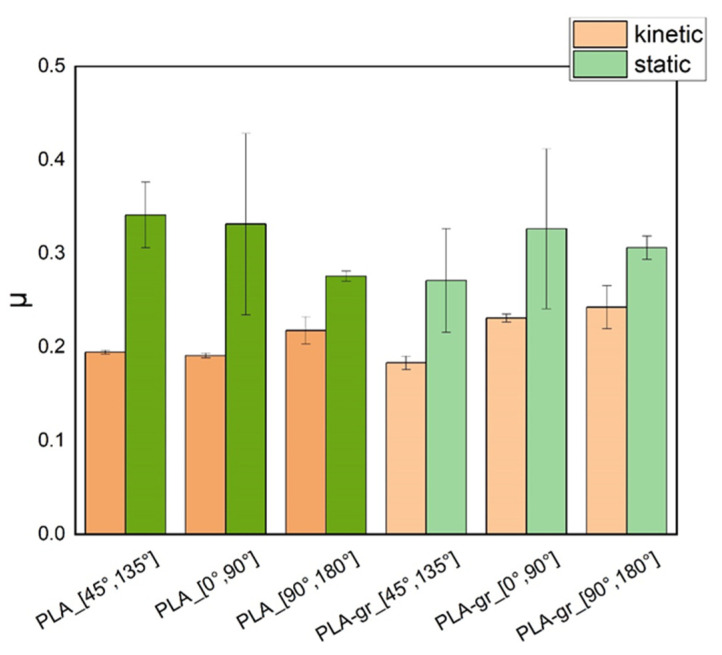
Graphical representation of the dependence of the friction coefficient, *μ*, of plain structures bearing only printer-induced surface micropatterning on the pattern orientation.

For plain structure samples carrying only micropatterns due to different manufacturing orientations, the evolution of the friction coefficient is plotted versus tip distance travelled on the samples, for pure PLA and PLA–graphene materials, in Figure 10 and Figure 11, respectively. The static friction coefficient corresponds to the first peak (maximum) of each diagram, while the kinetic friction coefficient was obtained as the average value of the succeeding part of the curve after the initial peak, i.e., after the sample had started moving on the plate. Averaging was employed in order to compensate for the considerable evidenced noise. Static and kinetic friction coefficients were calculated from the corresponding parts of the curves, for the plain structure samples made from pure and composite material and for the three different orientations of printer-induced micropatterning.

The mean values of the respective coefficients for three measurements at each micropatterning orientation are presented in the column plot of Figure 11, and the data are presented in tabular form in Table 2. It can be observed that for pure PLA, the [0°, 90°] orientation exhibits the lowest kinetic friction coefficient, a finding attributed to the parallelity between the pattern microlines and the sample’s direction of motion on the plate. Τhe [45°, 135°] orientation shows moderate friction, while the [90°, 180°] orientation exhibits the highest coefficient, owing to the other extreme, the verticality of surface pattern microlines to the direction of motion. The trend is not retained in the nanocomposite samples where the lowest kinetic friction coefficient relates to the [45°, 135°] orientation. The finding probably attributes to printing precision due to which the micro-roughness of graphene affects the overall surface roughness of the sample. Overall, the static and kinetic friction coefficients for pure PLA, ca. 0.3 and ca. 0.2, compare favorably with the literature [24,25].

**Figure 12:**
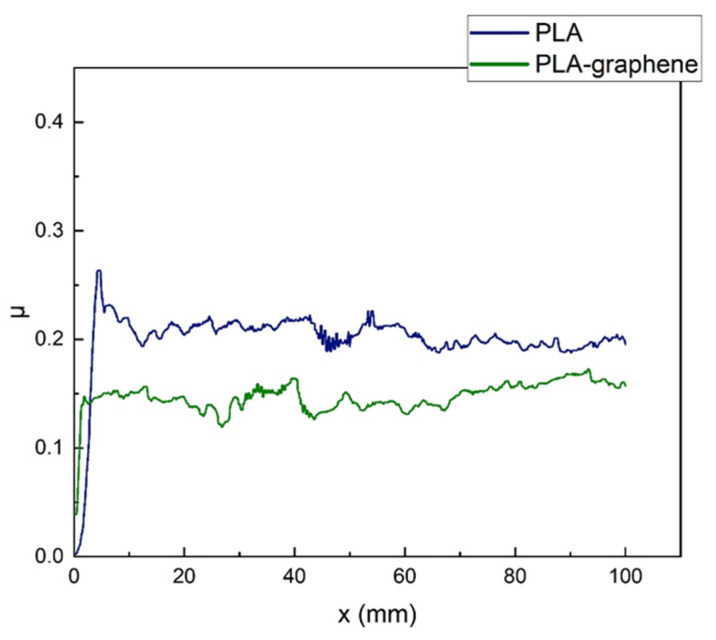
Evolution of the coefficient of friction, *μ*, with distance travelled on sample for 3D circular geometric patterns made of pure PLA (solid blue line) and composite PLA–graphene nanocomposites (solid green line).

**Figure 13:**
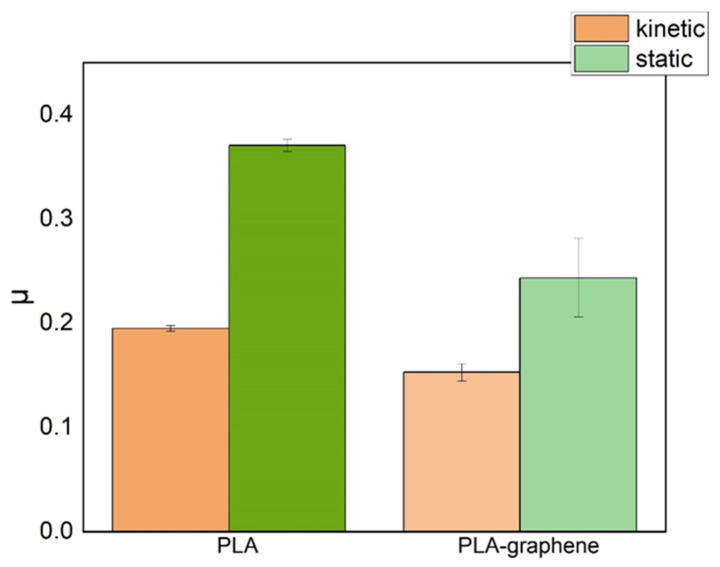
Graphical representation of mean value of the friction coefficient, *μ*, for samples with 3D-printed circular geometric patterns.

The temporal evolution of the friction coefficient on samples with 3D circular geometric patterning is shown in the diagrams of Figure 12, while the mean values of coefficients are tabulated in Table 3 and plotted in Figure 13. It is observed that graphene-doped samples (PLA-GNP) exhibit systematically lower friction coefficients than pure-PLA ones; a corresponding 21.5% decrease in the kinetic friction coefficient and a 34.5% decrease in the static one, compared with pure polymeric samples, are recorded. It is important to highlight that graphene presence appears to have a more significant effect on the friction coefficient of geometrically patterned samples, which bear larger uniform surfaces as opposed to the micro-rough and wavy surface of samples with printer-induced micropatterns.

#### 3.4.2. Ball-on-Disk Tribometer

**Figure 14:**
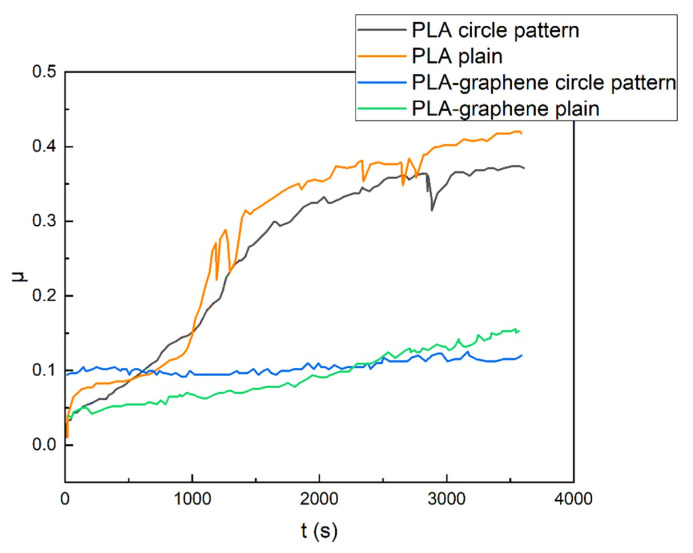
Temporal evolution of coefficient of friction for plain samples with printed-induced micropatterning (pure PLA, orange line, and composite, green line) and samples with 3D circular geometric patterning (PLA, gray line, and composite, blue line).

**Figure 15:**
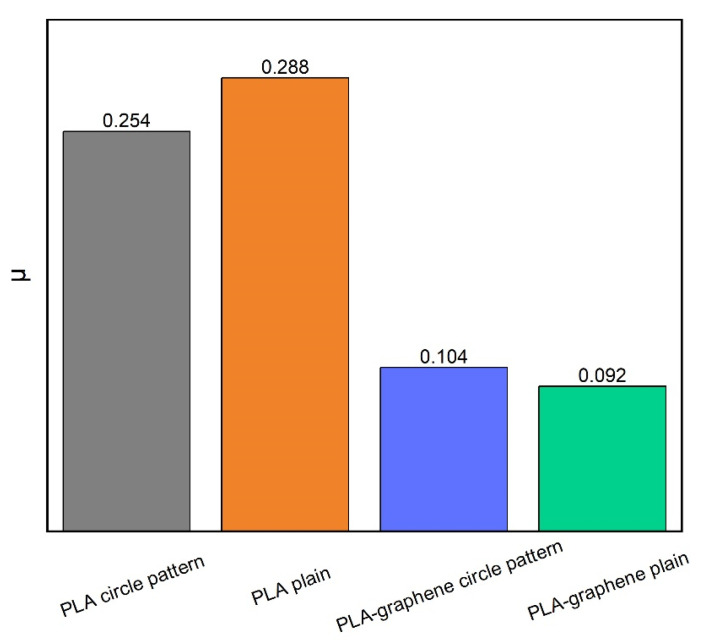
Graphical representation of the effect of graphene on the mean friction coefficient of PLA-based 3D-printed materials. On the LHS, pure PLA (plain samples, orange bar, and 3D-printed circular patterns, gray bar). On the RHS, corresponding values for graphene-doped counterparts (plain samples, green bar, and 3D-printed circular patterns, blue bar).

Based on the previous finding of the [0°, 90°] orientation as the one with the lowest kinetic friction coefficient, plain samples bearing printer-induced micropatterns of this orientation were tested on the commercial certified ball-on-disk tribometer, as did samples with 3D circular geometrical patterns. For each pattern type, both PLA and composite samples were examined. The temporal evolution of the friction coefficient is summarized in Figure 14 for all samples. The calculated mean values of kinetic friction coefficients are plotted schematically for all samples in the bar chart of Figure 15.

Significant reductions in the friction coefficient due to the presence of GNPs are observed. For the samples with 3D geometric circular patterns, a 59.1% decrease in the friction coefficient due to graphene is noted. The reduction reaches a record value of 68.1% for plain samples bearing only printed-induced micropatterning. Most impressively, the kinetic friction coefficient value of 0.092, achieved by graphene-doped samples is now of the same order of magnitude of the superlubric regime, a finding which highlights the major contribution of the nanomaterial to a tribological performance towards desirable superlubricity.

If the exclusive effect of geometric patterning on friction, rather than that of nanocarbon presence, was to be quantified, a comparison between plain samples bearing only printed-induced micropatterning and samples of the same material with 3D circular patterns would be of interest. This results in the finding of a 11.8% reduction in friction coefficient due to geometric patterning in pure PLA samples. The trend is slightly inversed in graphene-doped samples, with plain samples exhibiting coefficients which are by 13% higher than geometric samples. This slight divergence could possibly be attributed to defects in 3D-printed geometric patterns, which, in the presence of graphene nanoplatelets, impart additional surface roughness to the respective samples.

It is worth noting that the tribological properties measured using the conceptual purpose-built tribometer based on ASTM D1894 specs assume contact of the total surface area of the samples on a macroscopic level of characterization. In contrast, the ball-on-disk tribometer assumes a very small contact surface area of the ball with the sample, hence allowing microtribometric measurements. These considerations imply that individual graphene nanoplatelets resting on the yielded surfaces of each sample type, may give rise to different levels of contributions on their tribological behavior in each test. The differentiation may partially hinder direct comparison of the tribological results from the two measurement methods.

As a general result, in view tribological properties, current findings strengthen the allegation that polymers enhanced with graphene show advantages such as self-lubricating properties, lower weight, shock-dampening capability, and low noise [20,21]. At the same time, results clearly demonstrate the widening of applicability of PLA-based composites, at applications where tribological performance becomes essential. One example is biodegradable implants for load-bearing bone fractures or scaffolds, which opens up great opportunities in the field of bioengineering [22].

## 4. Conclusions

The combined effect of surface patterning and the presence of graphene inclusions on the tribological behavior of the most popular additive manufacturing material, PLA, was investigated. Plain samples bearing only printer-induced micropatterning and samples with 3D geometric patterns of circular columns were tested tribologically for the determination of the static and kinetic friction coefficients in both pure and graphene-doped polymer cases. Tests were conducted with two types of instrumentation, a purpose-built conceptual tribometer based on ASTM D1894 specifications and a certified commercial tribometer under the ball-on-disk configuration, to determine the effects of nanomaterial presence and patterning on the coefficients. Raman spectroscopic investigations established the multilayered nature, high degree of crystallinity, and absence of significant lattice defects of the nanoinclusions, whereas optical microscopy identified a non-homogeneous nanoplatelet distribution in the starting filament, a condition which SEM imaging did not detect in as-printed samples. Mechanical testing provided the required contact pressure for tribometric testing. The measured friction coefficients exhibited significant reductions due to graphene nanoplatelet presence, with values reaching a level of less than 0.1 and decreases of 68% for 3D-printed samples, highlighting an impressive potential of the nanocarbon for tribological performance towards the superlubric regime. Such reductions enable significant benefits in energy conservation, prolongation of equipment life, and protection of the environment, as envisaged by major sustainable future strategies. More research with other recyclable polymeric filaments and/or custom graphene dopings can also greatly assist the cause.

## Figures and Tables

**Table 1:** Main mechanical properties of tested samples.

Material	Manufacturing Method	Young’s Modulus (MPa)	Yield Strength (MPa)	Tensile Stress at Break (MPa)
Pure PLA	3D-printed	1446.1 ± 198.5	35.07 ± 5.31	32.31 ± 0.19
	Hot-pressed	1632.5 ± 102.2	41.59 ± 0.62	32.85 ± 2.89
PLA–graphene	3D-printed	1895.4 ± 200.1	34.74 ± 4.69	32.45 ± 3.82
	Hot-pressed	2195.9 ± 91.0	34.47 ± 1.60	33.56 ± 2.34

**Table 2:** Mean values of friction coefficients of samples bearing only printer-induced micropatterning of different orientations.

Material	Micropattern Orientation	Friction Coefficient	
		Kinetic	Static
Pure PLA	[45°, 135°]	0.195 ± 0.002	0.341 ± 0.035
	[0°, 90°]	0.191 ± 0.002	0.331 ± 0.097
	[90°, 180°]	0.218 ± 0.015	0.276 ± 0.006
PLA–graphene	[45°, 135°]	0.183 ± 0.007	0.271 ± 0.055
	[0°, 90°]	0.231 ± 0.004	0.326 ± 0.085
	[90°, 180°]	0.243 ± 0.023	0.306 ± 0.013

**Table 3:** Mean values of the friction coefficients of samples with the circle geometric pattern.

Material	Kinetic Friction Coefficient	Static Friction Coefficient
Pure PLA	0.195 ± 0.003	0.371 ± 0.006
PLA–graphene	0.153 ± 0.008	0.243 ± 0.038

## Data Availability

The original contributions presented in this study are included in the article. Further inquiries can be directed to the corresponding author.

## Abbreviations

The following abbreviations are used in this manuscript:

3D	three-dimensional
GNP	graphene nanoplatelets
PLA	polylactic acid
DLP	digital light processing
FDM	fused deposition modelling
SLM	selective laser melting
IJP	inkjet printing
LVDT	linear variable differential transformer

## References

[1] Holmberg K., Erdemir A. (2017). Influence of tribology on global energy consumption, costs and emissions. Friction.

[2] Nowak P., Kucharska K., Kaminski M. (2019). Ecological and Health Effects of Lubricant Oils Emitted into the Environment. Int. J. Environ. Res. Public Health.

[3] Ramezani M., Ripin Z.M., Jiang C.-P., Pasang T. (2023). Superlubricity of Materials: Progress, Potential, and Challenges. Materials.

[4] Hirano M., Shinjo K. (1993). Superlubricity and frictional anisotropy. Wear.

[5] Shekhar H., Dumpala R. (2021). Overcoming friction and steps towards superlubricity: A review of underlying mechanisms. Appl. Surf. Sci. Adv..

[6] Jang S., Kim S.H. (2023). Distinct effects of endogenous hydrogen content and exogenous hydrogen supply on superlubricity of diamond-like carbon. Carbon.

[7] Buzio R., Gerbi A., Bernini C., Repetto L., Vanossi A. (2021). Graphite superlubricity enabled by triboinduced nanocontacts. Carbon.

[8] Wang R., Zhang F., Yang K., Xiong Y., Tang J., Chen H., Duan M., Li Z., Zhang H., Xiong B. (2023). Review of two-dimensional nanomaterials in tribology: Recent developments, challenges and prospects. Adv. Colloid Interface Sci..

[9] Miura K., Ishikawa M. (2010). C60 Intercalated Graphite as Nanolubricants. Materials.

[10] Zhang J., Osloub E., Siddiqui F., Zhang W., Ragab T., Basaran C. (2019). Anisotropy of Graphene Nanoflake Diamond Interface Frictional Properties. Materials.

[11] Ge X., Chai Z., Shi Q., Liu Y., Tang J., Wang W. (2022). Liquid Superlubricity Enabled by the Synergy Effect of Graphene Oxide and Lithium Salts. Materials.

[12] Romanov R.I., Fominski D.V., Demin M.V., Gritskevich M.D., Doroshina N.V., Volkov V.S., Fominski V.Y. (2023). Tribological Properties of WS2 Thin Films Containing Graphite-like Carbon and Ni Interlayers. Materials.

[13] Bustillos J., Montero D., Nautiyal P., Loganathan A., Boesl B., Agarwal A. (2018). Integration of graphene in poly(lactic) acid by 3D printing to develop creep and wear-resistant hierarchical nanocomposites. Polym. Compos..

[14] Gkougkousi K., Karantzalis A.E., Nikolakopoulos P.G., Dassios K.G. (2024). Synergistic Effect of Carbon Micro/Nano-Fillers and Surface Patterning on the Superlubric Performance of 3D-Printed Structures. Materials.

[15] Rosenkranz A., Costa H.L., Baykara M.Z., Martini A. (2021). Synergetic effects of surface texturing and solid lubricants to tailor friction and wear—A review. Tribol. Int..

[16] Zhao Y., Mei H., Chang P., Chen C., Cheng L., Konstantinos G. (2021). Dassios. ACS Nano.

[17] Lin N., Li D., Zou J., Xie R., Wang Z., Tang B. (2018). Surface Texture-Based Surface Treatments on Ti6Al4V Titanium Alloys for Tribological and Biological Applications: A Mini Review. Materials.

[18] (2002). Standard Test Method for Tensile Properties of Plastics.

[19] (2023). Standard Test Method for Static and Kinetic Coefficients of Friction of Plastic Film and Sheeting.

[20] Ahmad A.F., Aziz S.A., Abbas Z., Obaiys S.J., Matori K.A., Zaid M.H.M., Raad H.K., Aliyu U.S. (2019). Chemically reduced graphene oxide-reinforced poly (lactic acid)/poly (ethylene glycol) nanocomposites: Preparation, characterization, and applications in electromagnetic interference shielding. Polymers.

[21] Butt J., Bhaskar R., Mohaghegh V. (2022). Non-destructive and destructive testing to analyse the effects of processing parameters on the tensile and flexural properties of FFF-printed graphene-enhanced PLA. J. Compos. Sci..

[22] Laraba S.R., Rezzoug A., Avcu E., Luo W., Halimi R., Wei J., Li Y. (2023). Enhancing the tribological performance of PLA-based biocomposites reinforced with graphene oxide. Biomed. Mater..

[23] Wu F.-B., Zhou S.-J., Ouyang J.-H., Wang S.-Q., Chen L. (2024). Structural Superlubricity of Two-Dimensional Materials: Mechanisms, Properties, Influencing Factors, and Applications. Lubricants.

[24] Cardoso P.H.M., de Oliveira M.F.L., de Oliveira M.G., Thire R.M.d.S.M. (2020). 3D Printed Parts of Polylactic Acid Reinforced with Carbon Black and Alumina Nanofillers for Tribological Applications. Macromol. Symp..

[25] Stoimenova N., Kandevab M., Zagorskib M., Paneva P. (2024). Static and Kinetic Friction of 3D Printed Polymers and Composites. Tribol. Ind..

